# Stigma as a fundamental hindrance to the United States opioid overdose crisis response

**DOI:** 10.1371/journal.pmed.1002969

**Published:** 2019-11-26

**Authors:** Alexander C. Tsai, Mathew V. Kiang, Michael L. Barnett, Leo Beletsky, Katherine M. Keyes, Emma E. McGinty, Laramie R. Smith, Steffanie A. Strathdee, Sarah E. Wakeman, Atheendar S. Venkataramani

**Affiliations:** 1 Center for Global Health, Massachusetts General Hospital, Boston, Massachusetts, United States of America; 2 Harvard Medical School, Boston, Massachusetts, United States of America; 3 Mbarara University of Science and Technology, Mbarara, Uganda; 4 Center for Population Health Sciences, Stanford University School of Medicine, Stanford, California, United States of America; 5 Department of Health Policy and Management, Harvard T. H. Chan School of Public Health, Boston, Massachusetts, United States of America; 6 Division of General Internal Medicine and Primary Care, Brigham and Women’s Hospital, Boston, Massachusetts, United States of America; 7 Northeastern University School of Law, Boston, Massachusetts, United States of America; 8 Bouvé College of Health Sciences, Northeastern University, Boston, Massachusetts, United States of America; 9 Division of Infectious Diseases and Global Public Health, University of California at San Diego School of Medicine, San Diego, California, United States of America; 10 Mailman School of Public Health, Columbia University, New York City, New York, United States of America; 11 Department of Health Policy and Management, Johns Hopkins Bloomberg School of Public Health, Baltimore, Maryland, United States of America; 12 Department of Medicine, Massachusetts General Hospital, Boston, Massachusetts, United States of America; 13 Department of Medical Ethics and Health Policy, Perelman School of Medicine, University of Pennsylvania, Philadelphia, Pennsylvania, United States of America; 14 Leonard Davis Institute of Health Economics, University of Pennsylvania, Philadelphia, Pennsylvania, United States of America

## Abstract

Alexander Tsai and co-authors discuss the role of stigma in responses to the US opioid crisis.

**Summary points**

- The current United States opioid overdose crisis is a complex, multifaceted, public health emergency that urgently requires the implementation of evidence-based primary, secondary, and tertiary preventive interventions. We develop a typology of the stigma related to opioid use, showing how multiple dimensions of stigma continue to fundamentally hinder the response to the crisis.
- Public stigma is driven by stereotypes about people with opioid use disorders, such as their perceived dangerousness or perceived moral failings, which translate into negative attitudes toward people with opioid use disorders.
- Enacted stigma describes the behavioral manifestations of public stigma, including discrimination and social distancing. Public and enacted stigma, in turn, lead to delivery of suboptimal care and undermine access to treatment and harm reduction services.
- Public stigma and enacted stigma can become structural stigma when they become encoded in cultural norms, laws, and institutional policies. Collectively, these forms of stigma run at cross purposes to—and reduce public support for—public health–oriented policies to address the opioid overdose crisis.
- When people with opioid use disorders internalize or anticipate the public stigma attached to their illness, maladaptive behaviors (e.g., disengagement from care) leading to poorer health outcomes may occur.
- Each of these dimensions of stigma (structural, public, enacted, internalized, and anticipated) serve to reinforce each other, resulting in poorer health outcomes even as the epidemiology of opioid overdose mortality continues to change.
- These dimensions of stigma must be overcome to facilitate the requisite policy and programmatic changes needed to effectively address the opioid overdose crisis.

## Introduction

The current US opioid overdose crisis is a public health emergency. The age-adjusted mortality rate due to drug overdose more than tripled from 1999 to 2017 [1]. In recent years, the largest increases in drug overdose mortality were observed for drug overdose deaths involving nonpharmaceutical fentanyl, which increased by an average of 71% per year in 2013–2017 [1–4], whereas overdose deaths involving prescription opioids and heroin have remained steady [5]. Concomitant with the rapid expansion in the magnitude of the opioid overdose crisis is an expansion in its scope, with increasingly syndemic [6] involvement of cocaine and psychostimulants [7–10].

There are multiple, interrelated, and deeply rooted social and economic determinants of the US opioid overdose crisis, none of which are likely to provide a sufficient explanation for the crisis when considered in isolation [11–17]. Although the epidemiology of opioid use and opioid use disorders (OUDs) has changed over time, the stigma attached to opioid use has endured [18]. Stigma is defined as a process wherein people with a particular social identity are labeled, stereotyped, and devalued, unfolding within the context of unequal and often preexisting power relations, leading to discriminatory behavior against people with the stigmatized identity [19–21] (**Box 1**). Its persistence and its persistent effects on health are consistent with its conceptualization as a “fundamental cause” of population health inequities across multiple social and physical health outcomes [22,23]. In the following discussion, we describe how these multiple dimensions of stigma are a fundamental hindrance to the response to the US opioid crisis.

**Box 1. Typology of the stigma related to opioid use.**

- Public stigma [24] is driven by stereotypes about people with OUDs [18], such as their perceived dangerousness [25,26] or perceived moral failings [27], which translate into negative attitudes.
- Anticipated stigma occurs when people with a stigmatized identity are subjectively aware of such negative attitudes and develop expectations of being rejected were their stigmatized (and potentially hidden) identities to become known [28]. Among people who do not have the stigmatized identity, perceived stigma refers to subjective awareness of negative attitudes and expectations of rejection were a hypothetical stigmatized identity to become known.
- Internalized stigma results when people with a stigmatized identity come to accept their devalued status as valid, thereby adopting for themselves the prevailing negative attitudes embedded in public stigma [29,30].
- Enacted stigma describes the behavioral manifestations of public stigma, including discrimination and social distancing [31,32].
- Family members and friends may experience all of these forms of stigma as a result of their affiliation with people with OUDs, a phenomenon described as courtesy stigma [21].
- Structural stigma refers to the totality of ways in which societies constrain those with stigmatized identities through mutually reinforcing institutions, norms, policies, and resources [33–36].

## The US opioid overdose crisis response: Multiple levels of prevention needed

Given the complexity of the US opioid overdose crisis, an effective response will require a multicomponent portfolio of public health, social, and economic policy interventions to address its social and structural determinants [11–17]. Health system interventions will also be necessary to implement primary, secondary, and tertiary prevention of opioid overdoses [37]. First, exposure to opioid use among opioid-naive patients should be minimized (primary prevention). Second, among those exposed to opioids, nonmedical use of prescription opioids and the incidence of OUDs must be reduced (secondary prevention). Third, expanded evidence-based treatment for OUDs is needed so that people with existing OUDs can achieve sustained remission, while the harms of ongoing opioid use (e.g., overdose) must be reduced for people who cannot, or do not choose to, achieve sustained remission (tertiary prevention). In combination, these preventive interventions will address what Humphreys and Pollack [38] have referred to as the “stock” and “flow” of the crisis: treatment and harm reduction for people already diagnosed with OUDs while simultaneously reducing the number of people with new diagnoses.

One component of primary and secondary prevention is promoting cautious and thoughtful opioid prescribing. People who are exposed to opioids—including prescribed opioids—are at increased risk of long-term use and of developing OUDs [39–41]. Surveys of people with OUDs often identify prescription opioids as the initiating opioid [42]. Thus, the importance of the admonition to “keep opioid-naive patients opioid naive” (p. 1454) [43] cannot be overstated. Other strategies to support primary and secondary prevention have been discussed extensively: reducing incautious and long-term opioid prescribing; preventing diversion; and identifying patients who may be at risk for, or who have already developed, OUDs [44–46].

Given current trends, however, prescribing-focused interventions alone will be insufficient to address the crisis. Opioid prescribing has already been in a nearly decade-long decline [47–49]. There is also evidence that the incidence of initial opioid prescriptions for opioid-naive patients has declined [50]. Despite these favorable trends, drug overdose mortality has continued to increase [1], with nonpharmaceutical fentanyl and its analogues increasingly associated with drug overdose deaths [1–5]. Thus, a singular focus on physician prescribing of opioids, to the exclusion of other prevention efforts, is unlikely to improve outcomes for those engaging in nonmedical use of opioids (e.g., nonpharmaceutical fentanyl) [51,52].

Tertiary prevention should focus on expanding evidence-based treatment of OUDs and reducing the harms of ongoing opioid use. Gold-standard, first-line treatment of OUDs consists of opioid agonist medication (methadone) or partial agonist medication (buprenorphine) [53]. Psychosocial interventions can also be effective when offered in conjunction with medication [54]. Yet only a minority of people with OUDs receive treatment of any kind, even after nonfatal overdose [55,56]. This underuse represents a missed public health opportunity, given the well-established effectiveness of opioid agonist treatment in reducing mortality [55,57]. Evidence-based harm reduction strategies for people with refractory OUDs include access to sterile injection equipment to reduce secondary transmission of HIV and hepatitis C [58–60], supervised consumption facilities [61] and supervised treatment with diacetylmorphine (heroin) [62] to reduce overdose risk, and expansion of overdose education and naloxone distribution to reduce the case-fatality rate of opioid overdoses when they do occur [63–65].

## Stigma as a fundamental hindrance to the US opioid overdose crisis response

### Public and enacted stigma

Negative attitudes toward people with OUDs undermine secondary prevention responses. People whose chronic noncancer pain syndromes have led to their physiological dependence on prescription opioid medications may be marked with the same labels as people with OUDs (e.g., morally weak, “addicts”) and experience difficulties obtaining care [66,67]. Healthcare professionals’ stigmatizing beliefs [68] can lead to provision of suboptimal care, a form of enacted stigma that reduces patients’ engagement with drug treatment [69–71]. In some instances, these suboptimal care patterns may extend to maintaining overly rigid and nonbeneficent care policies, lacking respect for patient autonomy, and deploying punitive care terminations in response to policy violations (e.g., smoking) or positive urine toxicology screening [66,70]. In the 3 years since the US Centers for Disease Control and Prevention published its new opioid-prescribing guideline [72], stigma enactments have included the imposition of rigid dosage or duration caps or initiation of noncollaborative tapers with established patients, escalating potential harms and transition to nonprescription opioids [73–78]. Enacted stigma has also been directly associated with nonfatal overdose [79].

Tertiary prevention is compromised when the stigma attached to OUDs affects access to treatment and harm reduction services. Among physicians who have obtained the US Drug Enforcement Agency (DEA) waiver to prescribe buprenorphine [80], most are not prescribing at capacity [81–83]; of these, many express little interest in taking on patients to reach capacity [84]. This gap in treatment access may reflect provider distancing from patients stereotyped as “difficult,” dangerous, or being involved in criminalized behaviors [85,86]. Other providers may wish to avoid the legal scrutiny associated with being a considered a “pill mill” (e.g., DEA audits) [87] or to avoid the courtesy stigma attached to caring for patients with OUDs [86,88]. Stigma has also undermined the wider distribution of overdose education and naloxone in the community. Specific challenges include anticipated stigma [89], concerns about moral hazard [90–92], or ignorance about state legislation related to naloxone prescribing or dispensing [93,94]. Most states have passed “Good Samaritan laws” to limit criminal liability for bystanders who provide or summon aid when witnessing an overdose incident [95], but the street-level effectiveness of such legislation may be limited by anemic implementation and punitive signaling by criminal law and law enforcement, including high-profile “drug-induced homicide” prosecutions, loss of housing, and other legal repercussions [96,97].

### Structural stigma

Public and enacted stigma can become structural stigma when they become encoded in cultural norms, laws, and institutional policies. The effects of structural stigma on undermining treatment of OUDs can be observed in how care is financed and delivered. Treatment is generally covered by state Medicaid programs [98], but prior authorization requirements and arbitrary lifetime treatment limits impose significant barriers to care [99–102], and many physicians who have obtained the waiver to prescribe buprenorphine do not accept third-party payments [103–105]. The 2010 Patient Protection and Affordable Care Act, among its many functions, sought to transform substance use treatment financing and delivery by mandating that effective treatments for substance use disorders be covered by third-party payers and integrated into mainstream healthcare systems [106,107]. However, the Affordable Care Act remains under threat, with attendant implications for the US opioid overdose crisis response [108]. Even where treatment is covered, treatment availability is commonly undermined by exclusionary zoning practices (that are themselves the product of public stigma [109,110]), further eroding engagement in care [111]. Concerns about treatment access are exacerbated in rural settings [112].

Other stigmatizing polices affect the health of people with OUDs in ways that are unrelated to treatment. Laws criminalizing the possession and distribution of certain substances codify stigma through both normative and instrumental pathways [97]. Many transplant centers consider OUDs, chronic opioid use, or opioid agonist treatment to be a relative contraindication to proceeding with transplantation [113–115]. Similarly, people with a history of injection drug use, or people on opioid agonist treatment (irrespective of injection drug use history), are routinely excluded from receiving post-acute care in skilled nursing facilities [116] or parenteral antimicrobial therapy in outpatient settings [117,118]. Physicians identified as having OUDs who are required to enroll in physician health programs are often required to adhere to abstinence-only approaches and discontinue opioid agonist treatment as a condition of maintaining professional licensure [119]. Taken together, these types of policies and related decision-making not only reinforce the ways in which people with OUDs are treated separately from others but also implicitly classify people with OUDs as being unworthy of investment and undeserving of treatment—thereby potentially having direct effects on health outcomes.

More generally, the language used to frame the crisis can influence norms about OUDs and about people with OUDs among policymakers and their constituents, directly affecting the policy levers that are brought to bear on the US opioid overdose crisis response. Everyday use of stigmatizing language negatively influences attributions of responsibility and increases support for punitive judgments (e.g., “substance abuser” [120]) or devalues evidence-based treatment of OUDs (e.g., “medication-assisted treatment” [121]). News coverage of the US opioid overdose crisis has inadequately emphasized treatment [122,123] and has instead largely framed the crisis as a criminal justice issue [124], particularly in ways that are racially disparate [125]. The very use of the moniker “epidemic” to describe the overdose crisis invokes isolation, quarantine, vector control, and other measures befitting infectious disease control but that are poorly calibrated for responding to the multifactorial opioid overdose crisis [97]. Language matters, because the judicious use of frames can shift public stigma [122,123], which can then become encoded structurally in the lack of public support for public health–oriented policies to address the opioid overdose crisis [126–131].

### Internalized and anticipated stigma

When people with OUDs internalize or anticipate the public stigma attached to their illness, maladaptive behaviors leading to poorer health outcomes may occur [132,133]. Among people with OUDs, internalized stigma has been associated with psychological distress and poorer quality of life [134–136], continued substance use [137], and reduced engagement with substance use treatment [138]. Anticipated stigma has also been associated with psychological distress [135,139] and reduced engagement in care [71,140–142]. In one nationally representative study of patients attending Health Resources and Services Administration health centers, stigma was one of the most commonly reported reasons for not engaging in substance use treatment [143]. The effects of internalized and anticipated stigma on treatment engagement can be especially pronounced in rural areas and small communities where treatment providers and their staff have multiple or overlapping relationships, thereby heightening concerns about boundary violations or breaches of confidentiality [144].

For people with OUDs who do initiate treatment, continued adherence and retention in care are needed to optimize outcomes. Longitudinal studies have consistently found that long-term retention in buprenorphine treatment is poor, with only one-third retained in care at 2 years [145–147]. No studies have directly linked stigma to retention in care for opioid agonist treatment, but among persons with other stigmatized conditions like HIV, stigma has been found to be a consistently strong correlate of treatment adherence, retention in care, and treatment outcomes [148,149].

Finally, the organization of treatment delivery itself can be stigmatizing to patients. Opioid treatment programs providing methadone—and the supervision, monitoring, and restrictive dispensing policies involved—are often experienced as degrading and humiliating [150–154]. Patient preferences for buprenorphine over methadone are routinely couched in terms of avoiding the stigma of methadone use [151,155]. These aspects of treatment delivery are themselves a product of stigma (i.e., are informed by negative stereotypes applied to people with OUDs) and are not necessary features of treatment itself [152].

### Considerations for health disparity populations

People with OUDs who are incarcerated or who have recently been released from incarceration have a particularly urgent need for evidence-based treatment. Detoxification during incarceration without linkage to treatment after release increases the risk of overdose substantially [156–158]. However, few receive treatment during incarceration or are linked to treatment after release [159–161]. This undertreatment of people with OUDs who also have a history of involvement with the criminal justice system, often motivated by stigma [162], represents a missed public health opportunity given the well-established effectiveness of opioid agonist treatment in reducing recidivism [163] and mortality [164–166].

One of the most pernicious examples of the interactions between structural stigma, public stigma, and enacted stigma can be observed in the racialized drivers of the US opioid overdose crisis and of the policy and programmatic response to the crisis. Because of racial disparities in receipt of opioid analgesia to treat acute pain [167] and treatment discontinuation following aberrant urine drug-test results [168], blacks (compared with whites) were comparatively less affected by the “second wave” of opioid-involved drug overdose deaths driven largely by the natural/semisynthetic prescription opioids being readily prescribed by physicians influenced by national clinical guidelines for assessment and treatment of pain [15]. However, with increasing nonmedical use of prescription opioids and use of nonprescription opioids among whites, news coverage about the opioid overdose crisis in suburban or rural white communities often features sympathetic, etiological narratives—whereas such accounts are typically missing in coverage of the opioid overdose crisis among blacks and Latinos [125]. This selective stigmatization evokes the racialization of crack cocaine, racially disparate federal prosecutions for crack (versus powder) cocaine, and disproportionately harsh federal sentencing of blacks that continue to have reverberating impacts in the present day [169,170]. The racialization of OUDs has resulted in a treatment and prevention approach among blacks that is characterized by overcriminalization and reliance on heavily regulated delivery of opioid agonist treatment segregated from traditional healthcare systems (i.e., daily dosing at community methadone clinics), whereas treatment and prevention among whites has become increasingly medicalized and addressed through office-based care [171,172]. In recent years, the burden of the opioid overdose crisis has disproportionately increased among blacks compared with whites [2]. Yet blacks receiving chronic prescription opioid treatment are more likely than whites to experience dose tapers [173], buprenorphine treatment remains largely concentrated among whites and in areas with higher proportions of white residents [174,175], and blacks who are able to access treatment are less likely to be successfully retained in care [146].

### Addressing stigma to support the US opioid overdose crisis response

The response to the US opioid overdose crisis urgently requires the implementation of evidence-based primary, secondary, and tertiary prevention. The policy changes that are needed to support these interventions are well understood and have been discussed extensively [80,176–178]. Yet the stigma attached to OUDs remains a fundamental hindrance to the crisis response. Multiple dimensions of stigma (structural, public, enacted, internalized, and anticipated) that are rooted in intersecting social categories serve to reinforce each other, resulting in poorer health outcomes even as the epidemiology of opioid overdose mortality continues to change. Stigma influences everyday attitudes, agenda setting, and policy-making. Stigma compromises the financing of care for OUDs, shapes the distribution of access to care, and impinges upon care delivery. Stigma even undermines the health of people with OUDs in ways that have nothing to do with the treatment of OUDs.

What is less well understood is how the stigma-related barriers to these requisite policy and programmatic changes can be overcome, either directly or indirectly. Direct interventions to reduce public and enacted stigma may take the form of persuasive communication or educational interventions—such as those deployed through mass media campaigns, law enforcement training [179], or schools—designed to improve understanding about the causes of and evidence-based treatments for OUDs. However, the sustained, long-term impacts of such interventions on stigma are unknown [180]. To improve care within healthcare delivery settings specifically, direct educational interventions may involve curricular changes at the level of undergraduate [181], graduate [182], or continuing [183] medical education. The specific educational content packaged within such interventions should be selected carefully. Education about the biomedical foundation of OUDs, for example, may not necessarily have the desired effects on stigma [184,185]. Contact interventions also hold promise for stigma reduction [186], but, as with educational interventions, their long-term effects remain unknown [187]. Finally, for people with OUDs, direct interventions to reduce internalized and anticipated stigma might draw on approaches from cognitive behavioral therapy [188] or acceptance and commitment therapy [189].

Indirect interventions might target institutions instead of individuals. For example, the mass media plays a central role in shaping our culture and therefore may represent a critical lever for influencing how multiple levels of stigma translate into specific behaviors and policies. Some print media organizations have issued professional guidance about reducing the use of stigmatizing frames in reporting about the opioid overdose crisis [190]. As another example, the “Changing the Narrative” project at Northeastern University (https://www.changingthenarrative.news) aims to reduce stigma and improve the accuracy of media portrayals, using various strategies such as promotion of style guides, “detailing” to journalists and editors, and social media monitoring and outreach. If disseminated nationally, similar to the Australian government’s support of the *Mindframe Media and Mental Health Project* [191], the impacts on public stigma could be substantial [123,126,129,192,193]. Some academic journals [194,195] and academic societies [196] have adopted similar guidelines. Broadcast media, rather than conditioning people to be fearful of persons with OUDs [197], can potentially reduce public stigma by portraying people with OUDs in ways that provide viewers with insight into the structural factors surrounding their use of opioids. However, as with print media, guidance on film and television portrayals fall under the purview of multiple trade groups and companies, and the levers for intervention are likely to be similarly diffuse.

The extent to which these direct or indirect interventions will successfully translate into changes in structural stigma, including agenda setting and policy change, remains unknown. Elected officials’ voting behavior and successful policy enactment are contingent upon numerous factors [198–200], of which public stigma is only one. Moreover, the systematic disenfranchisement of people with past criminal convictions is one of the most spiteful barriers to policy reform, because it strips voting rights from people whose behaviors were criminalized by the very laws that they are now powerless to change [97]. Thus, there will remain a need for civil society organizations and activist initiatives to have a central role in influencing governmental responses to the overdose crisis. More research is needed to understand the causal pathways through which stigma acts so that interventions targeting these mechanisms can be deployed to enhance the opioid overdose crisis response. Until the stigma of OUDs is addressed, it will continue to hinder implementation of these interventions, exacerbating existing population health inequalities.

## Abbreviations

DEA: US Drug Enforcement Agency
OUD: opioid use disorder
